# Comparative transcriptomic analyzes of human lung epithelial cells infected with wild-type SARS-CoV-2 and its variant with a 12-bp missing in the E gene

**DOI:** 10.3389/fmicb.2022.1079764

**Published:** 2023-01-09

**Authors:** Yi-Sheng Sun, Hao Sun, Han-Ping Zhu, Gao-Lei Li, Fang Xu, Hang-Jing Lu, An Tang, Bei-Bei Wu, Yu-Dong Li, Ping-Ping Yao, Jian-Min Jiang

**Affiliations:** ^1^Key Laboratory of Vaccine, Prevention and Control of Infectious Disease of Zhejiang Province, Zhejiang Provincial Center for Disease Control and Prevention, Hangzhou, China; ^2^Department of Biological Engineering, School of Food Science and Biotechnology, Zhejiang Gongshang University, Hangzhou, China; ^3^Key Laboratory of Health Risk Factors for Seafood of Zhejiang Province, Zhoushan Municipal Center for Disease Control and Prevention, Zhoushan, Zhejiang, China

**Keywords:** COVID-19, RNA-seq, lncRNAs, E gene, immune response

## Abstract

The severe acute respiratory syndrome coronavirus 2 (SARS-CoV-2) is a novel coronavirus that caused a global outbreak of coronavirus disease 2019 (COVID-19) pandemic. To elucidate the mechanism of SARS-CoV-2 replication and immunogenicity, we performed a comparative transcriptome profile of mRNA and long non-coding RNAs (lncRNAs) in human lung epithelial cells infected with the SARS-CoV-2 wild-type strain (8X) and the variant with a 12-bp deletion in the E gene (F8). In total, 3,966 differentially expressed genes (DEGs) and 110 differentially expressed lncRNA (DE-lncRNA) candidates were identified. Of these, 94 DEGs and 32 DE-lncRNAs were found between samples infected with F8 and 8X. Gene Ontology (GO) and Kyoto Encyclopedia of Genes and Genomes (KEGG) analyzes revealed that pathways such as the TNF signaling pathway and viral protein interaction with cytokine and cytokine receptor were involved. Furthermore, we constructed a lncRNA-protein-coding gene co-expression interaction network. The KEGG analysis of the co-expressed genes showed that these differentially expressed lncRNAs were enriched in pathways related to the immune response, which might explain the different replication and immunogenicity properties of the 8X and F8 strains. These results provide a useful resource for studying the pathogenesis of SARS-CoV-2 variants.

## Introduction

The novel coronavirus disease 2019 (COVID-19) is an acute respiratory infectious disease caused by a severe acute respiratory syndrome coronavirus 2 (SARS-CoV-2) infection, which was first identified in China (Zhu et al., 2020). All the human beings are generally susceptible to the SARS-CoV-2 virus. By the 4th of December (Kim et al., 2022), it had led to more than 640 million patients and more than 6.6 million deaths worldwide (WHO, 2022). The SARS-CoV-2 virus is a single-stranded RNA virus and can mutate easily. Since the outbreak of COVID-19 in the late of 2019, the SARS-CoV-2 virus has continuously evolved with many variants emerging across the world, such as Alpha, Beta, Delta and Omicron variants (Cao et al., 2022; Iketani et al., 2022). In particular, the Omicron variants BA.4 and BA.5, containing the prominent immune escape characteristics, have spread all over the world and become the dominant strains currently (Cao et al., 2022). In China, a large part of the clustered outbreaks and sporadic cases were caused by the BA.5 infection (Feng et al., 2022; Jiang et al., 2022), which placed great pressure on COVID-19 prevention strategies. Therefore, it is necessary to pay more attention to SARS-CoV-2 variants.

The envelope (E) protein, located at the viral envelope, is the smallest one of four major structural proteins of SARS-CoV-2 virus. It consists of only 76 amino acids, but plays important roles in the viral life cycle, such as viral assembly, budding and so on (Castano-Rodriguez et al., 2018). A SARS-CoV strain lacking the E protein is attenuated *in vivo* (Regla-Nava et al., 2015). In our previous study, we isolated both the E gene wild-type SARS-CoV-2 strain 8X and the E gene mutant strain F8 from the same specimen (Sun et al., 2020). Although no significant difference in the viral titer and infectivity was found in the E gene mutant SARS-CoV-2 strain F8 and the E gene wild-type strain 8X, F8, which contains a 12-bp deletion in the E gene, could produce a higher S protein content and induce a quicker humoral immune response than 8X. The inactivated SARS-CoV-2 vaccine produced from the F8 strain could trigger higher levels of the IgG titer and neutralizing antibody titer than those from the 8X strain at 1 and 3 weeks post vaccination, respectively. It seemed that the E gene mutation could influence the replication and immunogenicity of SARS-CoV-2. However, the mechanism has not been elucidated yet.

In this study, the whole-transcriptome sequencing was performed based on the F8-and 8X-infected human lung epithelial (Calu-3) cells. Subsequently, differentially expressed genes (DEGs) and lncRNAs of the F8 and 8X groups were analyzed, followed by functional interaction prediction analysis. Although large amounts of data have proven that several lncRNAs are involved in different kinds of viral infections, the underlying mechanisms by which they act are still largely unknown. Based on the whole-transcriptome sequencing analysis, our results may provide novel insights into the molecular basis of SARS-CoV-2 infection.

## Materials and methods

### Ethics statement

All the experiments related to live SARS-CoV-2 viruses were approved by the Ethics Committee of the Zhejiang Provincial Center for Disease Control and Prevention (ZJCDC) in China, and carried out in the biosafety level 3 (BSL-3) laboratory of ZJCDC.

### Virus and cells

The SARS-CoV-2 clinical strains F8 and 8X were purified from the pharyngeal swab of a male COVID-19 patient in Hangzhou as mentioned previously (Yao et al., 2020). Calu-3 cells were obtained from the National Collection of Authenticated Cell Cultures and cultured in DMEM (Gibco, United States) with 10% fetal bovine serum (FBS, Every Green, China) at 37°C in a 5% CO_2_ incubator. Cells were seeded into 6-well plates at a density of 1*10^6^ cells/well, and were infected by the F8 and 8X strains at a multiplicity of infection (MOI) of 2. Phosphate-buffered saline (PBS) was used as a negative control. Each group had two replicates. One hour post-adsorption at 37°C, the viral inocula were discarded, and the cells were maintained in the virus growth medium (DMEM containing 3% FBS) after washing twice with PBS. Two days post-infection, cells were collected, and the total RNA was extracted by using an RNeasy Plus Mini Kit.

### RNA extraction and next-generation sequencing

The RNA concentrations and quality of each sample were measured using Nanodrop One. The RNA integrity was detected by Agilent 2,100. Total RNA was used by depleting ribosomal RNA according to the manuscript of the Ribo-Zero rRNA Removal Kit. The rRNA-depleted RNA was fragmented, and the cDNA library was constructed using the TruSeq RNA sample Prep Kit (Illumina, San Diego, CA, USA). The sequencing libraries were sequenced on an Illumina NovaseqTM 6,000 platform according to the manufacturer’s instructions. The sequence data generated from this project has been deposited in NCBI under SRA submission PRJNA909976.

### RNA-Seq data analysis

Transcript assembly: First, Cutadapt (Martin, 2011) was used to remove the reads that contained adaptor contamination, low-quality bases and undetermined bases. Then, sequence quality was verified using FastQC (Babraham Bioinformatics, 2022). We used Bowtie2 (Langmead and Salzberg, 2012) and Hisat2 (Kim et al., 2015) to map reads to the genome of Homo sapiens. The mapped reads of each sample were assembled using StringTie (Pertea et al., 2015). Then, all transcriptomes from three samples were merged to reconstruct a comprehensive transcriptome using Perl scripts. After the final transcriptome was generated, StringTie and edgeR (Robinson et al., 2010) were used to estimate the expression levels of all transcripts.

LncRNA identification: First, transcripts that overlapped with known mRNAs and transcripts shorter than 200 bp were discarded. Then, we utilized CPC (Kong et al., 2007) and CNCI (Sun et al., 2013) to predict transcripts with coding potential. All transcripts with CPC scores < −1 and CNCI scores <0 were removed. The remaining transcripts were considered as lncRNAs.

Differential expression analysis of mRNAs and lncRNAs: StringTie was used to determine the expression level for mRNAs and lncRNAs by calculating FPKM (Trapnell et al., 2010). The differentially expressed mRNAs and lncRNAs were selected with log2 (fold change) >1 or log2 (fold change) < −1 and with statistical significance (I value < 0.05) by R package – edgeR (Robinson et al., 2010).

Target gene prediction and functional analysis of lncRNAs: To explore the function of lncRNAs, we predicted the cis-target genes of lncRNAs. LncRNAs may play a cis role by acting on neighboring target genes. In this study, coding genes in 100,000 upstream and downstream were selected by python script. Furthermore, trans-regulation analysis is a genome-wide search for well-associated target genes. Correlation analysis was performed between lncRNAs and the corresponding gene set. Associations with Pearson correlation coefficients greater than 0.4 (*p* < 0.05) were presumed to have targeted regulatory effects. Then, functional analysis of the target genes for lncRNAs was performed by using the BLAST2GO (Conesa et al., 2005).

### Go and KEGG enrichment analysis

Gene Ontology (GO) enrichment analysis of differentially expressed genes or lncRNA target genes was conducted with respect to biological process, molecular function, and cellular component. Kyoto Encyclopedia of Genes and Genomes (KEGG) was used to perform pathway enrichment analysis. The R package clusterprofiler was used to perform the detailed enrichment analysis described above (Wu et al., 2021).

### Construction of the LncRNA-protein-coding gene co-expression network and competing endogenous RNA (ceRNA) network

For each lncRNA, the Pearson correlation coefficient of its expression value with that of each protein-coding gene was calculated. Under the conditions of an absolute value of the Pearson correlation coefficient > 0.90 and *p* < 0.001, the interaction network of the differentially expressed lncRNAs and protein-coding gene co-expression pairs was then constructed using Cytoscape (Shannon et al., 2003).

When constructing the competing endogenous RNA (ceRNA) network, lncRNAs were connected to the differentially expressed (DE) human miRNAs if they were predicted to interact with each other, and the upregulated (downregulated) miRNAs were connected to downregulated (upregulated) mRNAs if the former targeted the latter based on the database miRTarBase (Huang et al., 2020). The lncRNA-miRNA-mRNA network was visualized using Cytoscape (version 3.9.1). The co-expression network was built as follows: A custom database from a combination of public databases, starBase (version 2.0) and miRcode (version 11), was used to predict the interaction between known lncRNAs and miRNAs. The interaction of miRNA with novel lncRNA, circRNA, and mRNA was performed by using TargetScan (version 8.0) and miRanda (version 22.1), using default parameters. Pairs that appeared in both results were considered as prospective interactions. Subsequently, correlation analysis was performed between ceRNAs (circRNA, lncRNA, mRNA) and miRNAs. Based on the correlation between them, a hypergeometric distribution analysis was performed under the threshold of probability less than 0.05. The construction of the ceRNA network followed the following principles: (1) Pearson correlation coefficient within ceRNA pairs or between ceRNAs and miRNAs should be under the threshold of an absolute value of 0.4 (*p* < 0.05); and (2) At least one miRNA must satisfy the hypergeometric distribution test between two ceRNAs.

### Statistical analysis

All the statistical analyzes in this study were conducted in R (version 4.2.1). The Wilcoxon rank-sum test was used to compare the sample means between different groups, and was conducted with the wilcox.test function. Significance was expressed as a *p* value < 0.05.

## Results

### Transcriptome profiles of SARS-CoV-2 infected cells

**Figure 1:**
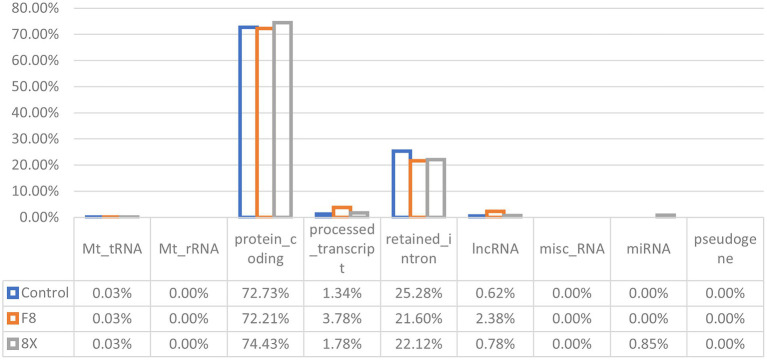
RNA categories of quantified transcripts in RNA-seq. The percentages of each RNA type are shown in F8/8X infected or mock-infected Calu-3 cells.

To identify different transcripts in SARS-CoV-2 infected cells, the transcriptomes of the Calu-3 cells infected with F8, 8X, or without SARS-CoV-2 infection as controls were detected using high-throughput RNA sequencing. Robust and reproducible data were obtained from all samples. After quality filtering, clean reads were mapped to the human reference genome (hg38) using HiSat2, and were assembled with StringTie. Then, coverage analysis was performed on these clean reads on different annotated gene types. The distribution of each type of gene was counted according to the expression level (Figure 1). In total, eight categories of RNA were identified according to database annotation of those transcripts, in which the protein-coding genes were highly represented (72.21% in F8 and 74.43% in 8X, respectively).

### Identification of differentially expressed genes

**Figure 2:**
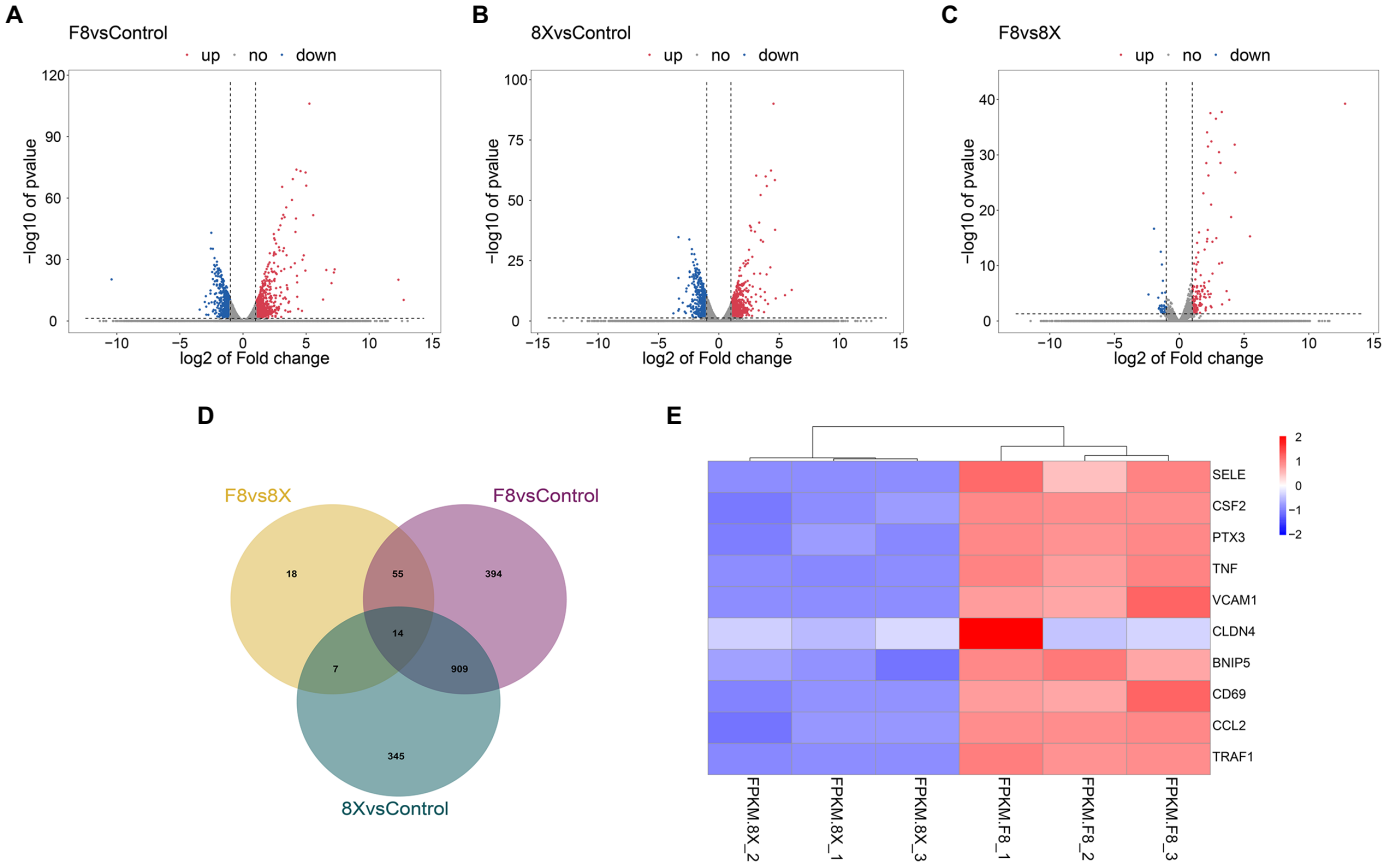
Volcano plots showing the DEGs between 8X and control **(A)**, F8 and control **(B)**, and the 8X and F8 **(C)**. **(D)** Venn diagram of the identified DEGs shared among the above three groups. **(E)** Heatmap of the top 10 DEGs between 8X and F8 groups.

DEGs were identified from the control vs. SARS-CoV-2 infection groups and used for functional annotation. All DEGs were demonstrated in volcano plots (Figures 2A–C). In the F8 vs. 8X group, there were 149 upregulated DEGs and 41 downregulated DEGs. The comparison between the F8 vs. control or 8X vs. control groups revealed that a total of 1,372 or 1,275 genes were significantly regulated after viral infection. A Venn diagram was plotted to identify the common or unique DEGs in human lung cells with or without SARS-CoV-2 infection (Figure 2D and Support file 2). A total of 14 common DEGs were identified. Given the exposure of cells to F8, the number of DEGs was smaller (345 genes vs. 394 genes) in comparison of the 8X vs. control group. The top 10 DEGs, including the E-selectin (SELE), colony stimulating factor 2 (CSF2) and pentraxin 3 (PTX3), were shown in the heatmap (Figure 2E).

### Go/KEGG analysis of DEGs

**Figure 3:**
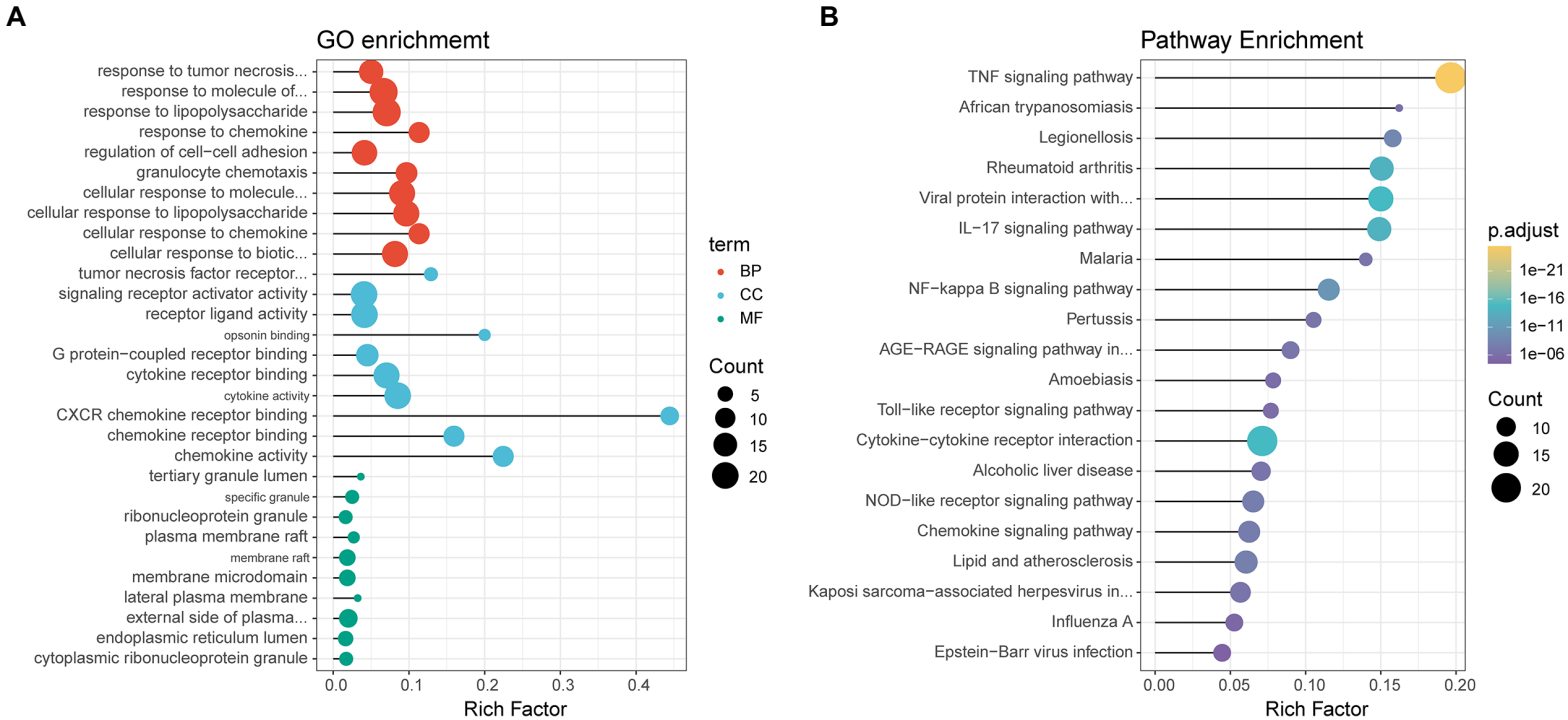
Dot plots displaying enriched GO terms **(A)** or KEGG pathways **(B)** of identified DEGs. Dot size represents the count of the enriched gene within each category, and the x-axis represents the gene ratio (rich factor). The GO terms/KEGG pathways are arranged on the basis of the value of the gene ratio, not on their adjusted *p* value, for easier visualization purposes.

GO enrichment of the DEGs was divided into three types: biological process, cellular component, and molecular function (Figure 3A). Each top ten terms of the three types, such as cytokine activity and membrane raft, were displayed. The top 20 enriched pathways were shown through KEGG functional analysis of the DEGs (Figure 3B). Significant enrichment in the TNF signaling pathway, viral protein interaction with cytokine and cytokine receptor, and cytokine-cytokine receptor interaction were found.

### Identification of lncRNAs and Go/KEGG analysis of lncRNA target genes

**Figure 4:**
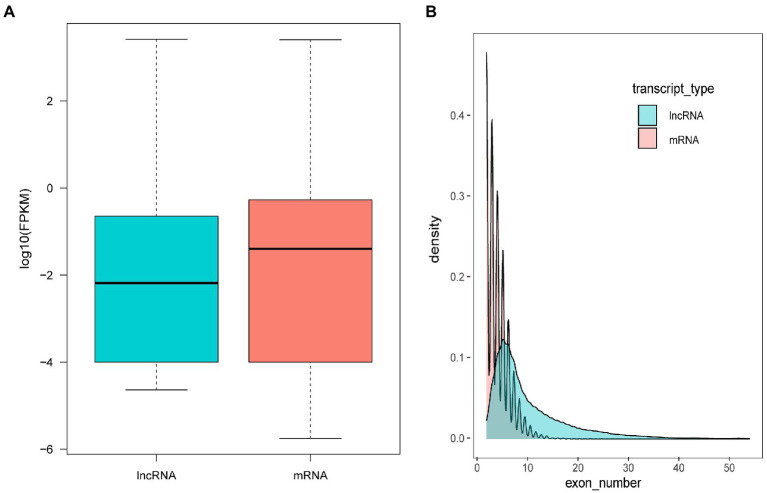
Comparison of the expression level **(A)** and exon number **(B)** of lncRNAs and protein-coding genes.

The assembled transcripts were used to identify lncRNAs using the pipeline as described in the Methods. In total, 110 annotated and novel lncRNAs were identified. It has been reported that lncRNAs, in comparison with protein-coding genes, usually share some common genomic features with their sequences. However, they are generally shorter in length, have fewer but longer exons, and have lower expression levels and lower evolutionary sequence conservation (Liu et al., 2019). To further determine the characteristics of the lncRNAs identified in the present study, we compared transcript length, exon number and expression levels of protein-coding genes and lncRNAs. The results revealed that fewer exons and lower expression levels were also found in the lncRNAs, which was consistent with the reported lncRNAs (Figure 4).

**Figure 5:**
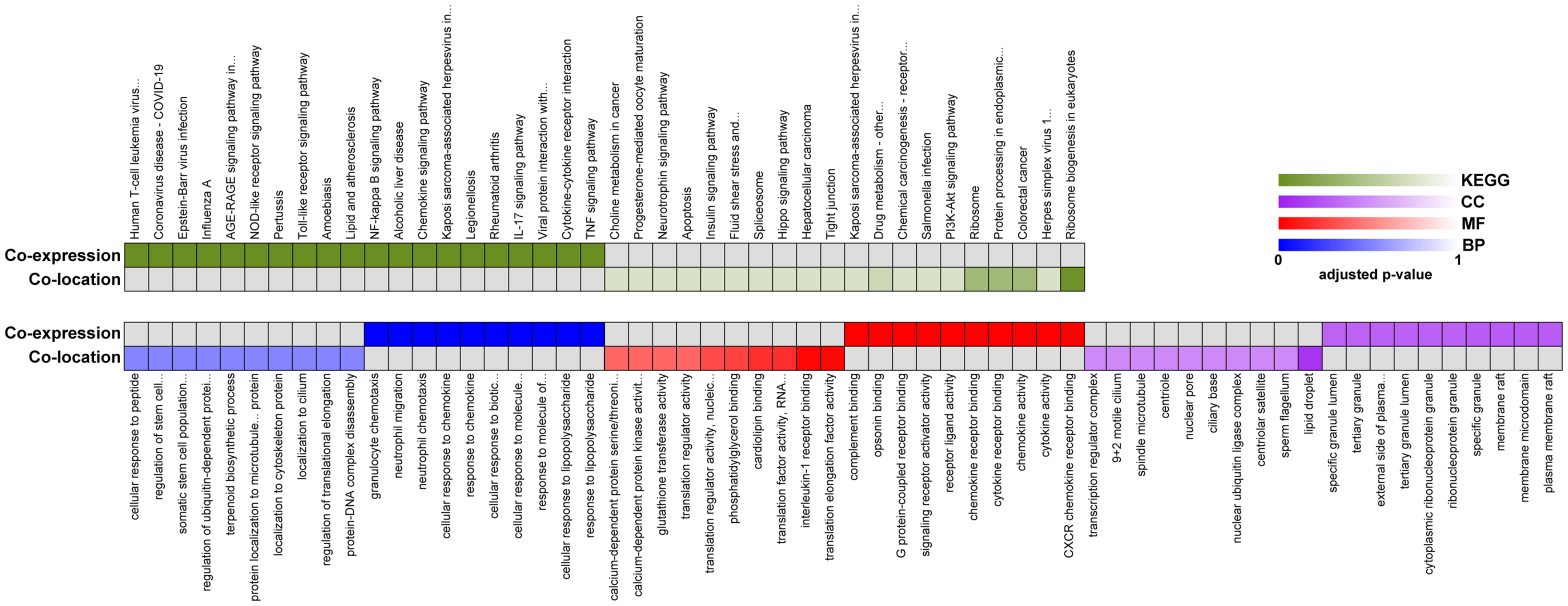
Functional enrichment analysis of identified lncRNAs between 8X and F8. Representative over-represented KEGG pathway (top) and GO terms (bottom) of gene-expression clusters. BP, biological process; MF, molecular function; CC, cellular component.

We next performed GO and KEGG pathway analyzes for the target genes of lncRNAs between 8X and F8. The top 20 GO terms and KEGG pathways were reported in Figure 5. The GO term analysis divided differentially expressed lncRNAs into the same three types as DEGs. Biological processes such as response to chemokine and protein localization to cytoskeleton, the molecular functions such as cytokine activity and translation regulator activity, and the molecular functions such as membrane raft and transcription regulator complex, were all enriched. KEGG enrichment analysis revealed that pathways related to the immune system, such as viral protein interaction with cytokine and cytokine receptor, chemokine signaling pathway and Toll-like receptor signaling pathways, were preferentially targeted. Therefore, the results of GO and KEGG pathway enrichment analyzes revealed that lncRNAs may act in cis or trans to participate in the regulation of the expression of multiple important genes in different processes, including the immune response and protein localization.

### Expression correlation analysis

**Figure 6:**
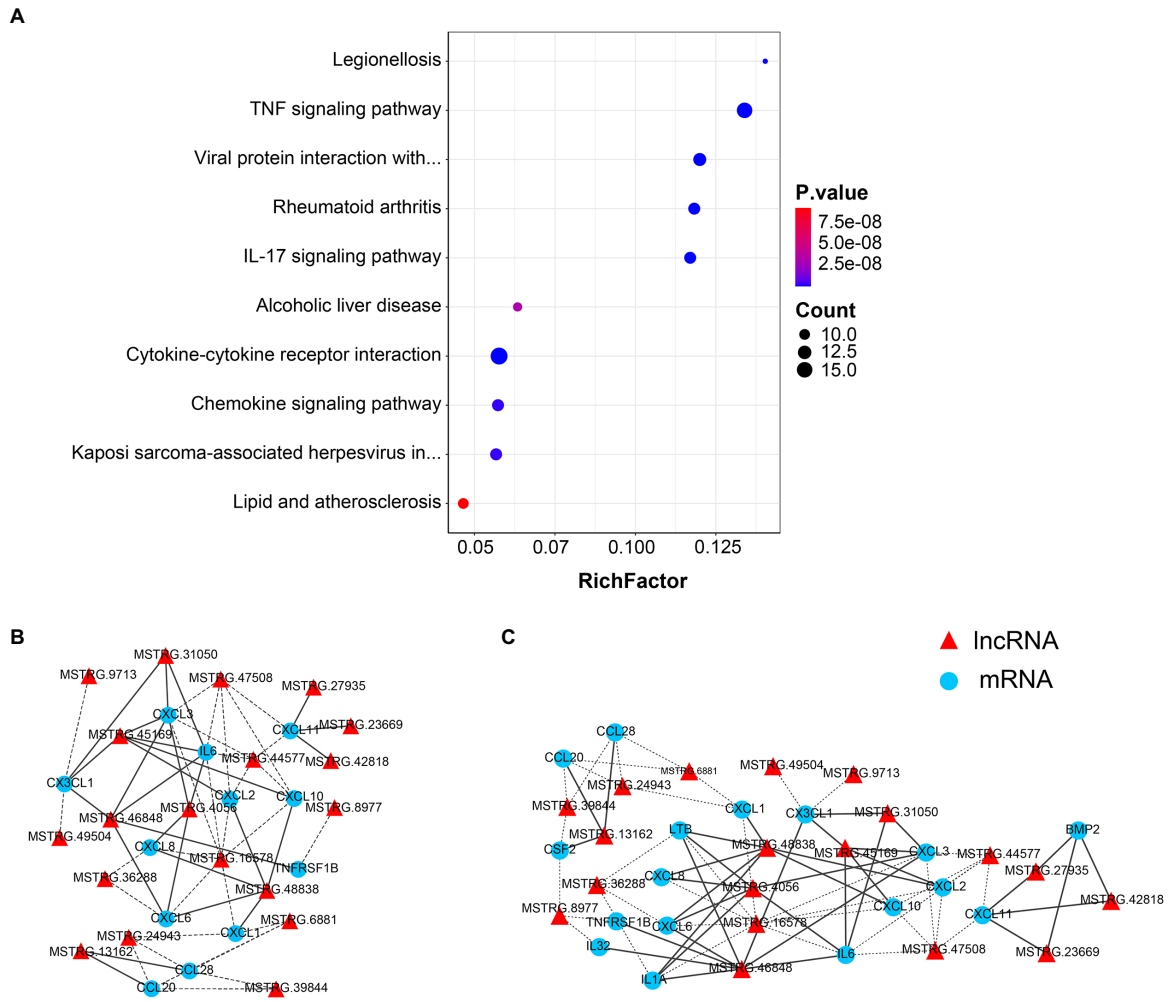
Co-expression analysis of lncRNAs and protein-coding genes. **(A)** The top 10 over-represented KEGG pathways of co-expressed genes. The network of lncRNAs with **(B)** viral protein interaction with cytokine and cytokine receptor, and **(C)** TNF signaling pathway-related genes (*p* < 0.01). The solid line represents a positive correlation, while the dotted line represents a negative correlation.

The functional association between regulatory lncRNAs and protein-coding gene transcripts can be determined by performing expression correlation analysis. To further investigate the potential mechanism of the SARS-CoV-2 associated lncRNAs, the DE lncRNAs and their predicted target DE protein-coding genes were investigated by delineating lncRNA-protein-coding gene functional interactions. Here we identified 325 pairs of DE lncRNA-DE protein-coding genes between 8X and F8 (Support file 1), containing 22 lncRNAs and 77 protein-coding genes (*p* < 0.01). The network of co-expressed lncRNA-protein-coding gene pairs based on a threshold of Pearson’s correlation coefficient of 0.998. Next, the KEGG pathway analysis of co-expressed genes revealed the top 10 pathways, including the TNF signaling pathway, viral protein interaction with cytokine and cytokine receptor, and cytokine-cytokine receptor interaction, which were all significantly enriched (Figure 6A).

The network of lncRNAs with viral protein interaction with cytokine and cytokine receptor (Figure 6B), and TNF signaling pathway-related genes (Figure 6C) were analyzed. Several common cytokines, such as IL-6, CXCL2 and CXCL10, were found in all these pathways. Moreover, the CSF2, one of the top ten DEGs between 8X and F8 groups, was also involved in both the viral protein interaction with cytokine and cytokine receptor pathway and the TNF signaling pathway.

### CeRNAs network analysis

**Figure 7:**
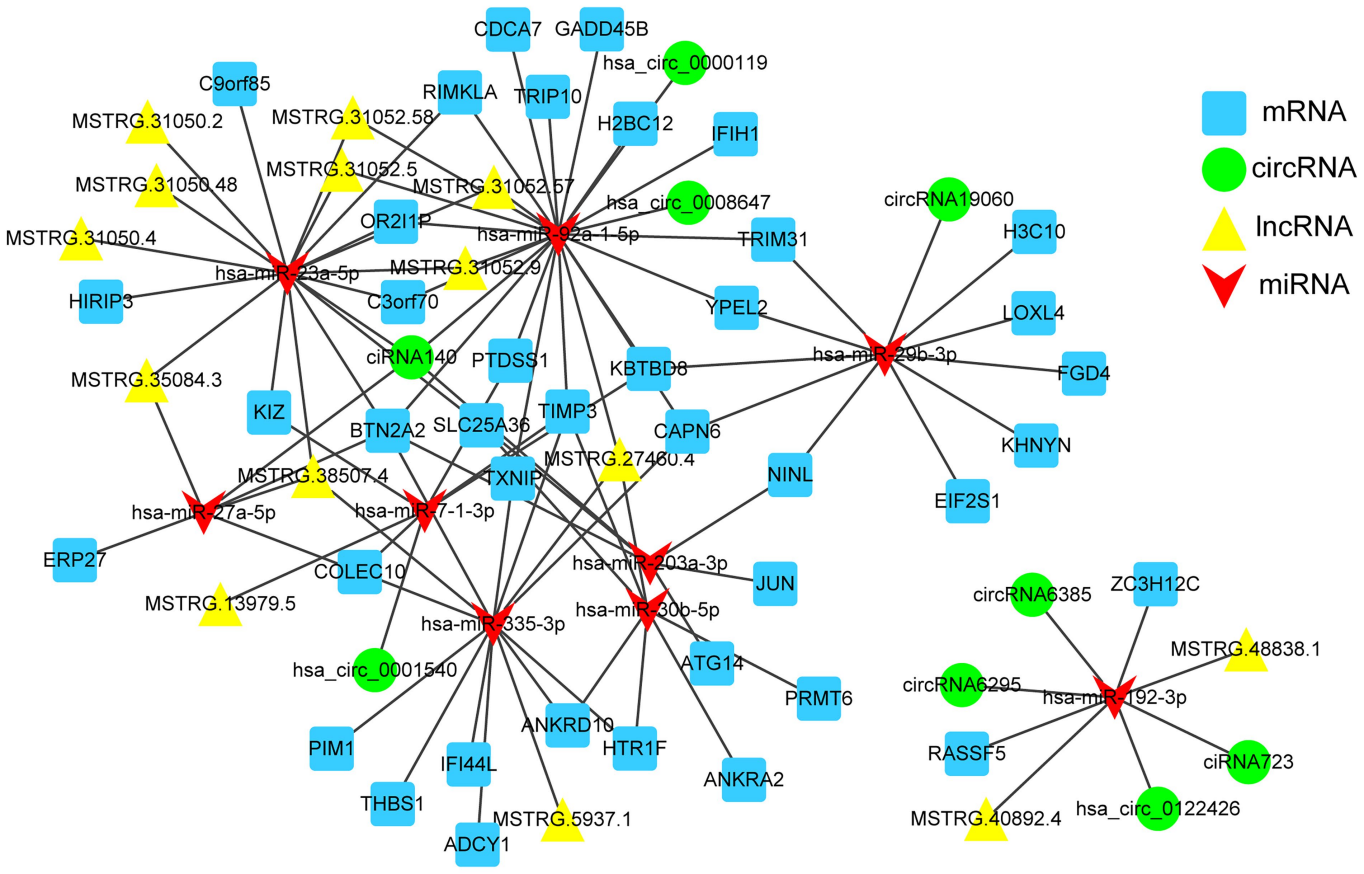
The competing endogenous RNA (ceRNA) network. mRNAs, circRNAs, host lncRNAs and miRNAs in the networks are represented as squares, circles, triangles and arrows, respectively.

Based on the lncRNA-mRNA co-expression relationship and the regulation relationship of DE-miRNA-DE-mRNA and DE-miRNA-DE-lncRNA, the lncRNAs and mRNAs that were significantly differentially expressed and regulated by the same miRNA were screened. In total, 89 lncRNA-miRNA-mRNA interactions were finally obtained. Furthermore, according to the lncRNA-miRNA-mRNA and circRNA-miRNA-mRNA networks, differentially expressed circRNAs, lncRNAs, and mRNAs that were regulated by the same miRNA were further screened. Finally, 101 interaction pairs were obtained (Figure 7). Among these interactions, there were 2 upregulated lncRNAs and 2 upregulated mRNAs, and 1 upregulated miRNA in Calu-3 cells infected with the F8 variant compared with the 8X strain. There were 12 lncRNAs, 25 upregulated and 11 downregulated mRNAs, and 1 upregulated and 7 downregulated miRNAs in Calu-3 cells infected with F8 or X8 compared with the control.

## Discussion

Since the outbreak of COVID-19, a large number of studies have focused on the spike protein of SARS-CoV-2. However, few studies have focused on the E gene. Both the E gene and S gene are the structural proteins of SARS-CoV-2. E gene is important for the virus maturation and replication (Regla-Nava et al., 2015). Studies related to the E gene were mainly about its use as a target for the virus detection (Kim et al., 2022; Valadan et al., 2022). It seems that the E gene of SARS-CoV-2 is conserved. However, in our previous study (Sun et al., 2020), we found that a 12-bp deletion in the E gene of the F8 variant was more likely generated as a result of viral adaptation to Vero cells. The E gene of SARS-CoV-2 might have several mutations. Since the E gene mutant strain F8 and E gene wild-type strain 8X were both from the same specimen, these two strains had similar genetic backgrounds. It would be interesting to use them to investigate the function of the E gene.

In this study, by applying transcriptome sequencing, we found 3,966 DEGs and 110 differentially expressed lncRNAs in Calu-3 cells infected with F8 compared with 8X. Direct functionly enrichment analysis of the DEGs showed that these genes were mainly involved in cytokines and chemokines, which are related to the host immune response. What’s more, the co-expression analysis of differentially expressed lncRNAs and DEGs also showed the enrichment of the pathways involved in immune response, which may be critical for viral maturation, such as the TNF signaling pathway, and viral protein interaction with cytokine and cytokine receptor. A previous study showed that lncRNAs play a key role during viral infection (Liu et al., 2019). Additionally, some lncRNAs and miRNAs, such as hsa-miR-335-3p, hsa-miR-92a-1-5p, and hsa-miR-23a-5p, were identified as hub genes in the ceRNA network. Hsa-miR-92a-1-5p was found to enhance the interferon expression by targeting the suppressors of cytokine signaling 5 to inhibit feline panleukopenia virus replication in host cells (Liang et al., 2022). All the results strongly suggest that the genes involved in the immune response, especially the cytokines and chemokines, may play fundamental roles in the pathogenesis of different SARS-CoV-2 variants. Further study might be carried out in the SARS-CoV-2 E gene variant-infected animal models.

Through analysis of DEGs between the F8 and 8X groups, 85 upregulated genes were found. The E-selectin, CSF2 and PTX3 were the top 3 of the most upregulated genes. The E-selectin is important for leukocyte accumulation in inflammatory responses and PTX3 can amplify the immune response (Aslan et al., 2012; Ketter et al., 2016). In our previous research, a different immunogenicity of the inactivated COVID-19 vaccine produced by the F8 strain was found compared with that produced by the 8X strain (Sun et al., 2020). F8 vaccine might induce a higher expression level of E-selectin and PTX3 than 8X vaccine to promote a quicker humoral immune response in mice. The TNF-α signaling pathway is also important for the immune response, and is one of the most upregulated pathways in SARS-CoV-2 infected A549-hACE2 cells (Sun et al., 2021). The release of TNF-α could trigger several cell adhesion molecules, such as selectins and VCAM-1, to induce inflammation *in vivo* (Kong et al., 2018), which is a protective biological response for eliminating viruses. In our study, the TNF signaling pathway was also enriched significantly through the KEGG functional analysis of DEGs, the co-expression correction analysis of regulatory lncRNAs, and protein-coding gene transcripts. The expression levels of TNF in the F8 group were 24.3-fold higher than those in the 8X group. It seems that the TNF signaling pathway is also related to the pathogenesis of different SARS-CoV-2 variants.

Cytokines and chemokines are important for the immune response (Berantini et al., 2022). Among the viral protein interaction pathway, the immune system pathway and the TNF signaling pathway, cytokines such as IL-6 and CSF2, and the chemokines such as CXCL2 and CXCL10, were all involved. CXCL2 was also found to be one of the common DEGs in SARS-CoV-2 infected Calu-3, hCM and A549-hACE2 (both low and high viral loads) cells (Sun et al., 2021). IL-6 might be a reliable indicator for the COVID-19 severity, and a higher level of IL-6 concentration was found in severe COVID-19 patients than in the moderate or mild groups (Bergantini et al., 2022). The CSF2 protein was inhibited to reduce the production of inflammatory factors and chemotaxis of inflammatory cells (Xiong et al., 2022). CXCL10, also known as interferon gamma-induced protein 10 (IP-10), is a pro-inflammatory chemokine (Coperchini et al., 2020). Higher expression levels of IL-6, CSF2 and CXCL10 were also found in the F8 group than in the 8X group, indicating a more severe inflammatory response in the immunized mice of the F8 group.

In conclusion, our study systematically characterized transcriptome profiles during SARS-CoV-2 infection and provides a comprehensive genome-wide resource for identifying and functionally analyzing the differentially expressed genes and non-coding RNAs. Immune response signaling, such as upregulation of IL-6, CSF2 and CXCL10 cytokines and a higher level of E-selectin and PTX3, as well as the TNF-α signaling pathway, might be the reason to explain the different pathogenesis caused by the E gene mutant strain F8 and E gene wild-type strain 8X. Further functional validations are needed to delineate the exact mechanistic details, and an animal model might be used to study the pathogenesis of both the E gene mutant and wild-type strains. These results will be useful for better understanding the pathogenesis of SARS-CoV-2 variants, and designing better preventive and therapeutic measures against viral infection.

## Data availability statement

The data presented in the study are deposited in the NCBI repository, accession number: PRJNA909976.

## Ethics statement

The studies involving human participants were reviewed and approved by the Ethics Committee of the Zhejiang Provincial Center for Disease Control and Prevention (ZJCDC) in China. The patients/participants provided their written informed consent to participate in this study.

## Author contributions

Y-SS, HS, P-PY, and Y-DL conceived and designed the experiments. Y-SS, H-PZ, FX, H-JL, and AT performed the experiments. G-LL, HS, and J-MJ analyzed the data. Y-SS, J-MJ, and Y-DL drafted the manuscript. All authors read and approved the final manuscript.

## Funding

This work was supported by the Basic Public Welfare Research Project of Zhejiang Province (LGF22C080002, LGF22H260024, LDT23H19013H19), the Key R&D Program of Zhejiang Province (2021C03197, 2021C03200), and the Zhejiang Provincial Foundation for Scientific Research in Medicine and Health (WKJ-ZJ-2220).

## Conflict of interest

The authors declare that the research was conducted in the absence of any commercial or financial relationships that could be construed as a potential conflict of interest.

## Publisher’s note

All claims expressed in this article are solely those of the authors and do not necessarily represent those of their affiliated organizations, or those of the publisher, the editors and the reviewers. Any product that may be evaluated in this article, or claim that may be made by its manufacturer, is not guaranteed or endorsed by the publisher.
