# Supplementary figures and images for: Comparative transcriptomic analyzes of human lung epithelial cells infected with wild-type SARS-CoV-2 and its variant with a 12-bp missing in the E gene
Source: Front Microbiol. 2023 Jan 9;13:1079764. doi: 10.3389/fmicb.2022.1079764 (PMC9868179; doi:10.3389/fmicb.2022.1079764)

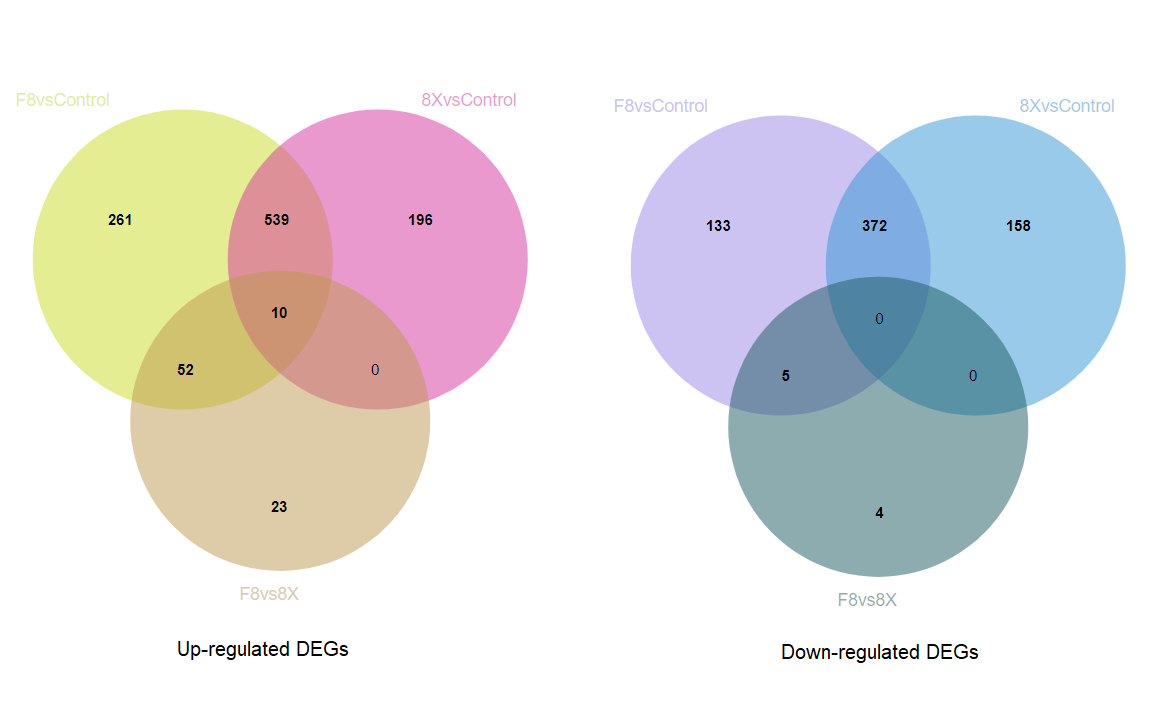

Supplement: Supplementary file 2 [file Image_1.PNG]
